# Report of a Case of Osteo-Lipo-Chondroma of the Upper End of the Humerus. Resection of Humerus. Exhibition of Patient

**Published:** 1895-07

**Authors:** Thomas R. Neilson

**Affiliations:** Surgeon to the Episcopal Hospital and to St. Christopher’s Hospital for Children


### REPORT OF A CASE OF OSTEO-LIPO-CHONDROMA OF THE UPPER END OF THE HUMERUS. RESECTION OF HUMERUS. EXHIBITION OF PATIENT.

BY THOMAS R. NEILSON, M.D.,

Surgeon to the Episcopal Hospital and to St. Christopher's Hospital for Children.

The case which I desire to report to the Academy is that of a young man who suffered from a tumor involving the head and upper end of the shaft of the right humerus. I saw him first in the early part of last February. His history was as follows: J. T. P., twenty-one years of age, worked on a farm until two years ago, when he learned the trade of electrical construction, in which his work has been light. Family history good; no history of tumors among relatives. In June, 1893, his attention was called by a fellow-workman, who saw him stripped, to an enlargement of the humerus, the size of a pigeon's egg. One year later it had slowly grown to twice that size. Afterward it grew more rapidly, and in November, 1894, it began to cause inconvenience by its size—limiting the motions of the joint, and giving rise to a sensation of numbness occupying the back of the arm as far down as the elbow; there was also loss of sensation in the fingers, and he noticed some diminution of power in the hand, especially in using the hammer. At no time was there any pain in the growth. There was no history of injury to the shoulder.

Upon examination, I found the upper end of the humerus markedly enlarged, the growth being hard and immovable, and having an irregular surface, the larger portion of it projecting inward toward the axilla, and preventing complete abduction of the arm. From the history, the slowness of the growth, the absence of pain, the irregularity of the surface, and the uniform density of the mass, I believed the tumor to be an enchondroma, and felt that resection was to be advised. In this opinion, Prof. Ashhurst, who kindly examined the patient for me, concurred.

The patient was admitted to the Episcopal hospital on February 7, 1895. The measurements of the right shoulder as compared with the opposite side were as follows: Around the most prominent part of the head of the bone, just below the acromion (arm adducted), right, 18 inches; left, 15 inches; two inches below the acromion (arm abducted) the circumference of the right shoulder was 14¾ inches, and of the left 12 inches.

*Operation.* On February 13, with the assistance of my colleagues, Drs. Harte and H. C. Deaver, I resected the growth by the deltoid flap operation. Starting my incision at the tip of the coracoid process, I carried it downward along the groove between the deltoid and pectoralis major, and then across the arm to the outer side at the level of the insertion of the deltoid. I next dissected back the skin and superficial fascia about an inch for the whole extent of the incision, and then divided the deltoid transversely about two inches above its insertion. The flap being turned back, the tendon of the long head of the biceps was freed from its groove and held to one side, the capsule of the joint opened, the insertions of the supra- and infra-spinatus and teres minor severed, and the attachments of the sub-scapularis divided. The growth was so large that it was found impossible to complete disarticulation until the attachments of the coraco-brachialis and of the pectoralis major were also divided. This being accomplished, I sawed through the humerus just above the insertion of the deltoid. After making the section I noticed a suspicious spot of degenerated tissue remaining, and, pushing back the periosteum with the insertion of the deltoid, I cut off another inch of the bone.

Throughout the operation there was no arterial hemorrhage of moment, only a few muscular branches requiring ligature; but the cephalic vein was, unfortunately, slightly torn at the beginning of the operation, while pushing it aside in the groove between the deltoid and the pectoralis major, and again later when delivering the growth. I tied it below the tear, and again some distance further up, and resected the intervening segment —about five inches in length.

This being done, the wound was flushed with normal salt solution, and the deltoid united with several quilted sutures of kangaroo tendon. A large rubber drainage-tube was inserted, and the wound closed with silk sutures. A bichloride-gauze dressing was applied and the arm bandaged to the side, a large axillary pad supporting it.

The patient did remarkably well and the wound healed kindly.

There was no noteworthy occurrence until the eighteenth day, when it was found that, at a point about the centre of the wound, just over the anterior axillary fold, the union had broken down, and there was a slight purulent discharge. The cause of this was found to be one of the kangaroo tendon sutures, which was working its way out. Four days later another of these sutures was discharged from the little sinus, and again in five days a third one. The sinus then healed firmly.

On March 30th the patient was discharged from the hospital. At that time the contraction of the deltoid showed that it had united firmly, in spite of the fact that three of the kangaroo sutures had cut their way out, owing to imperfect sterilization.

The patient, as you will observe, has now very good motion of the arm, and he tells me that he has returned to his former work at electrical construction. The specimen, which I present for your inspection, shows the tumor to be nearly twice the size of the fist, and to have grown largely toward the inner aspect of the bone. A section of the growth was examined by Dr. Joseph McFarland, pathologist to the Hospital, who reported the microscopical diagnosis to be osteo-lipo-chondroma.

